# Development of Small-Molecule Fluorescent Probes Targeting Enzymes

**DOI:** 10.3390/molecules27144501

**Published:** 2022-07-14

**Authors:** Yuan-Xiang Li, Dong-Tai Xie, Ya-Xi Yang, Zhao Chen, Wu-Yingzheng Guo, Wen-Chao Yang

**Affiliations:** 1 College of Chemistry and Materials Engineering, Huaihua University, Huaihua 418008, China; hhxyliyuanjun@163.com (Y.-X.L.); xdt15580137232@163.com (D.-T.X.); s200708052022@163.com (Y.-X.Y.); 2 Key Laboratory of Pesticide & Chemical Biology of Ministry of Education, International Joint Research Center for Intelligent Biosensor Technology and Health, College of Chemistry, Central China Normal University, Wuhan 430079, China; chenzhao@mails.ccnu.edu.cn (Z.C.); gwyz@mails.ccnu.edu.cn (W.-Y.G.)

**Keywords:** fluorescent probes, enzymes, biosensing, bioimaging

## Abstract

As biological catalysts, enzymes are vital in controlling numerous metabolic reactions. The regulation of enzymes in living cells and the amount present are indicators of the metabolic status of cell, whether in normal condition or disease. The small-molecule fluorescent probes are of interest because of their high sensitivity and selectivity, as well as their potential for automated detection. Fluorescent probes have been useful in targeting particular enzymes of interest such as proteases and caspases. However, it is difficult to develop an ideal fluorescent probe for versatile purposes. In the future, the design and synthesis of enzyme-targeting fluorescent probes will focus more on improving the selectivity, sensitivity, penetration ability and to couple the fluorescent probes with other available imaging molecules/technologies.

## 1. Introduction

Over the past decade, one can identify a trend towards research in drug discovery and chemical biology applications [1,2]. There has been wide research interest in chemical biology, namely, in developing small-molecule analogs to study biological functions, in order to control and to modify them. However, where the biological systems are concerned, the greater complexity and redundancy have made it difficult to conduct complete individual studies of natural biological molecules and the pathways they control. Therefore, attempts have been made to design small-molecule tools or chemical probes to study the roles of target molecules [3,4,5]. Compared with biomolecule (such as protein, peptide, and nucleic acids)-based imaging tools, the features of small-molecule fluorescent probes are obvious: low molecular weight, simple and non-natural structure. Due to these features, small-molecule fluorescent probes have the following advantages, low cost, easy synthesis and reserve, and high flexibility in probe design. More importantly, they usually cause minimal perturbation to the native target in a complex environment. Of note, there are some important issues that need to be addressed when developing small-molecule fluorescent probes. Their applications are seriously determined by those issues, such as solubility, cell penetration, biocompatibility and in vivo specificity. Enzymes are indispensable to many important life activities, and most of them are proteins in nature. Therefore, it is challenging to reveal the physiological functions of these enzymes by targeting these proteins. This type of probe can covalently bind to active sites of specific enzyme classes such as kinases, GTPases, and serine hydrolases. The probe can be used to study the target an enzyme or enzyme class, or specific inhibitors of the particular enzyme. The structure of the probes consists of an specific recognition group, a linker region, and a fluorophore [6,7]. All active site probes can be used to determine inhibition of enzymes by small molecules. Additionally, some probes preferentially react only with active enzymes, allowing for activity-based proteomic profiling (ABPP) [8]. A recent advance is that the probe could be designed so that it has a fluorophore, allowing the detection to be more easily automated and, at the same time, more accurate and precise. In designing fluorescent probes, the highest possible absolute detection at the level of single molecules is expected [9,10,11]. The review focuses on the discovery and application of fluorescent probes targeting enzymes. 

## 2. Design Principles of Fluorescent Probes

A successful fluorescence probe should be noninvasive (not invading healthy tissue), imaging stable (stable under physiological conditions and not subject to photo-bleaching), selective (ability to target an analyte of interest from its close analog) and sensitive (ability to exhibit strong emission change upon interaction with a target). It should also possess a low limit of detection (LOD) and a dynamic response range. If a probe is highly sensitive towards an analyte, it will have a low LOD. The wide response range is favorable when a mild-to-intense stage of an enzyme-catalyzed reaction or a similar metabolic process needs to be detected.

**Figure 1:**
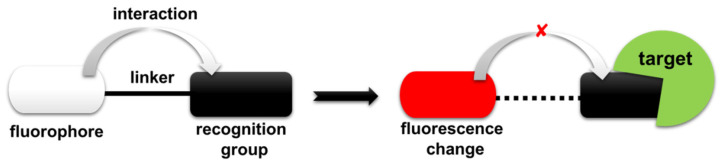
Design principle and sensing mechanism of fluorescent probes targeting enzymes.

Fluorescent probes targeting enzymes are small molecular compounds that can react specifically with specific enzymes and emit fluorescence. These compounds can accurately and rapidly bind or react with specific regions of targeted enzymes, so as to realize the monitoring function of fluorescent probes. Fluorescent probes can be applied to almost any type of cell [12,13]. Fluorescent probes are defined [14,15] as molecules that react specifically with biological molecules to break the interaction between fluorophore and recognition group and induce a concomitant change in their optical properties (fluorescence intensity, excitation/emission wavelength, etc.) (Figure 1). Current design strategies for fluorescent probes include photoinduced electron transfer (PET) [16], fluorescence resonance energy transfer (FRET) [17], intramolecular charge transfer (ICT) [18], excited-state intramolecular proton transfer (ESIPT) [19], aggregation-induced emission (AIE) [20], etc. Fluorescent probes are being widely used in identifying the enzymes, assaying enzyme activity, and studying the mechanism of action, screening of substrate/inhibitor activity, drug discovery and cellular imaging. Basically, the structure of the probes consists of three parts, a specific recognition group, a linker region, and a fluorophore [6,7] (Figure 1). Ideally, the fluorescence of the designed probe is quenched before interacting with the enzyme target and is recovered after binding with the enzyme target. When designing a fluorescent probe, the substrate structure, testing environment and also the optical requirement must be taken into consideration. In achieving the overall accurate result, it has to be noted that there is no single probe design applicable to all the enzymes, or even for one particular enzyme class [12,15]. Thus, we just chose some representative probes, as well as the probes reported by us.

The identified strengths of fluorescence as an imaging method are high sensitivity, low cost, high throughput, ease of performing, large number of available probes, and ability to monitor two or more probes simultaneously. Low resolution, low depth penetration, and limited *in vivo* specificity account for the weaknesses. The other available imaging methods are bioluminescence, Positron Emission Tomography (PET), Magnetic Resonance Imaging (MRI) and Single Photon Emission Tomography (SPECT).

## 3. Categorization of Fluorescent Probes

The fluorescent probes targeting enzymes could be broadly categorized in to two categories, active site based probes and active group based probes. Enzyme active site-based probes react with a particular class of enzymes based on the common conserved features in their catalytic domains. On the other hand, active group-based probes contain reactive groups which target multiple and diverse enzyme types, which could belong to different classes of enzymes [21]. Therefore, the specificity of the probes for single enzyme reaction needs to be improved.

### 3.1. Fluorescent Probes Targeting Serine Hydrolases

This class of enzymes catalyze the hydrolysis of specific covalent bonds such as ester bonds and amide bonds in particular substrates. Out of the hydrolase enzymes, serine hydrolase is one of the largest and most diverse eukaryotic enzyme families. Serine hydrolases are generally grouped into two large member families: serine proteases (e.g., trypsin, elastase and thrombin) and metabolic serine hydrolases. According to the substrate specificity of serine proteases, some high-selective fluorescent probes was developed for biosensing and bioimaging *in vitro* and *in vivo*.

**Figure 2:**
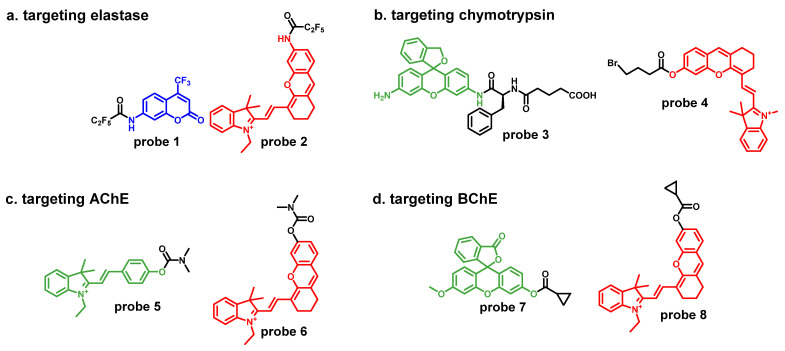
(**a**) The structures of **probes 1** and **2**; (**b**) The structures of **probes 3** and **4**; (**c**) the structures of **probes 5** and **6**; (**d**) The structures of **probes 7** and **8**.

Elastase is an important member of the serine hydrolase family and is involved in many important physiological processes, such as inflammation and immune response. Changes in elastase activity have also been associated with a variety of diseases, including acute lung injury and intestinal inflammation. Elastase can digest and decompose elastin in connective tissue proteins, including hydrolyzing peptide bond, amide, and ester, which can activate phosphatase A, reduce serum cholesterol, improve serum lipids, and lower plasma cholesterol [9,22]. Because pentafluoroethyl, as a recognition group, is well adapted to the active cavity of elastase, the amide bond of the probe is hydrolyzed by elastase and fluorophore is released, thus achieving the specific detection of elastase in aqueous solution and tumor mice model [23] (Figure 2a). **Probe 1** is the first non-peptide fluorescent probe targeting for elastase. It has excellent LOD: 68 ng/mL. However, its emission wavelength (505 nm) limits its application, and it is only applied *in vitro*. Near-infrared (NIR) fluorescence analysis has advantages in the detection of biological samples because of its strong penetration and low background signal [9,22]. Thus, **probe 2** was constructed based on the recognition group: pentafluoroethyl and the NIR fluorescence dye: hemicyanine. It shows high emission wavelength (700 nm), more excellent LOD (29.6 ng/mL) and strong *in vivo* detection ability. **Probe 2** can be used for imaging elastase in cell and mice models.

Chymotrypsin (Chy) is a common serine hydrolase, mainly involved in various physiological and biological events such as protein digestion, cell proliferation and apoptosis. It has been reported that abnormal expression of Chy can lead to a variety of diseases, such as pancreatic fibrosis, diabetes, indigestion, pancreatic cancer and so on. Therefore, the development of an effective method to monitor Chy activity in living systems can provide an important reference for the diagnosis, treatment and management of related diseases and cancers. Currently, a variety of fluorescent probes that can specifically recognize Chy have been developed, and the two probes shown in the figure can be applied at the cellular and *in vivo* levels (Figure 2b) [9,24,25]. **Probe 3** can be lightened by Chy or pancreatic juice, so it was developed for the detecting leakage of pancreatic juice during operation. **Probe 4** was NIR fluorescent probe for Chy, and it can image Chy in Kunming mice *via* skin-pop injection.

Acetylcholinesterase (AChE) plays an important role in many physiological processes such as cell differentiation, cell apoptosis and neural tissue development. Abnormal fluctuations in the AChE directly affect the metabolism of acetylcholine, thus interfering with neurotransmission in the brain. As a result, mood and behavior are inevitably affected and may even induce depression. Using a fluorescent probe as a tool is conducive to understanding the function of AChE and the treatment of related diseases. A number of highly specific fluorescent probes targeting AChE have been developed and successfully applied to *zebrafish* and mouse brain (Figure 2c) [26,27]. The recognition group (dimethylcarbamate) of **probe 5** was inspired by a famous AChE inhibitor, neostigmine. **Probe 5** was used to trace AChE in PC12 cells and the brains of mice. The structure of **probe 6** include the same recognition group as **probe 5** and a NIR fluorophore. It was applied in cell and zebrafish imaging. Notably, **probe 6** can clearly label the neuromast of *zebrafish*, which shows that **probe 6** has ultrahigh selectivity and spatial resolution.

Butyrylcholinesterase (BChE) is widely distributed in plasma, liver, muscle and brain tissues and is associated with lipid metabolism and various human diseases such as liver injury, diabetes, Alzheimer’s disease (AD) and liver metastasis. Therefore, using fluorescent probes to study and trace BChE is a good method. Yang and his colleagues developed a fluorescent probe with high sensitivity and specificity to evaluate BChE activity and inhibitor screening for high-throughput studies in drug discovery and clinical diagnosis (Figure 2d) [28]. The fluorescence probe developed by Liu and colleagues was successfully applied to AD model mice and detected the content of BChE in the brain of AD model mice (Figure 2d) [29].

### 3.2. Fluorescent Probes Targeting Proteases

**Figure 3:**
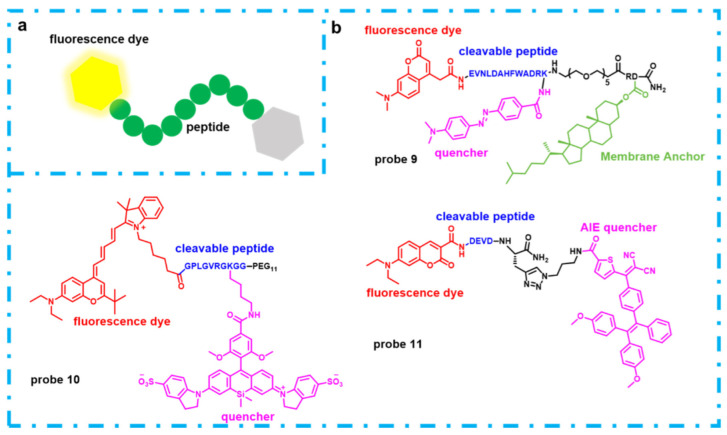
(**a**) The design strategy of the fluorescent probes for proteases; (**b**) Chemical structures and composition of **probes 9**, **10** and **11**.

Proteases are highly diversified enzymes in living cells and they are involved in breaking down of peptide substrates. However, aberrations of proteases lead to critical diseases including cancers, inflammation and neuro-degeneration [30]. In addition, some proteases from human, germs or virus were important drug target [31]. Therefore, many fluorescence probes were developed to respond to the demand of diagnose and inhibitor screening. Thanks to the fact that peptides are the substrate of proteases, the vast majority of protease-targeting fluorescent probes have a peptide-based structure. The kind of structure usually comprises a specific cleavable peptide and fluorescent dye-quencher pair to modify both ends of the peptide chain (Figure 3a). Based on the design strategy, many recent developments of fluorescent probes targeting proteases was reported. Some examples are exhibited in below. 

The aggregation of amyloid-β (Aβ) is a symptom and possible reason for Alzheimer’s disease. β-Secretase (BACE1) is the necessary protease in production of Aβ [32]. Therefore, it has become a potential target for Alzheimer’s disease. For monitoring the activity of BACE1 in Hela cells, **probe 9** was developed [33] (Figure 3b). Notably, the probe has a membrane anchor to help it to enter into the endosome, because BACE1 only shows activity in the endosome where the pH value is 4–5. 

MMPs (matrix-metalloproteinases) are calcium/zinc-dependent proteases and have 23 members. They are regarded as important extracellular matrix (ECM)-degradation enzymes. Because the concentration of ECM-degradation proteins in tumor and cancer microenvironments are always different from normal tissue, MMPs have a large potential as biomarkers for diagnosis of cancer and design of cancer imaging agents [34]. For example, **probe 10**, a fluorescence sensor for MMPs, comprises a peptide that can be recognized by MMP-2, MMP-9, MT1-MMP, and the novel dye-quencher pair [35] (Figure 3b). The probe can be used for imaging cancer in a mouse model of human fibrosarcoma.

Caspases, a family of proteases, play a crucial part in cell death and inflammatory reaction. Thus, developing a fluorescent probe can help us to understanding apoptosis and visual detecting inflammatory and cancer [36]. **Probe 11** was developed for detecting caspase-3, which is a subtype of caspases [37] (Figure 3b). Interestingly, the AIE (aggregation-induced emission) molecule is used as quencher in the probe. While the cleavable peptide of peptide is cut by caspase-3, the AIE molecule can aggregate to produce fluorescence emission. Thus, the probe can produce special dual-color fluorescence. 

### 3.3. Fluorescent Probes Targeting Oxidoreductases

As a large enzyme family, oxidoreductases catalyze the oxidation or reduction of substrates requiring electron donors or acceptors and take part in many physiological processes, such as metabolic activity, biosynthesis, energy production for organism, maintaining of redox homeostasis and so on [38]. These enzymes widely exist in many tissues and organs of living body, including nitroreductase (NTR), monoamine oxidase, quinone oxidoreductase and so on. 

**Figure 4:**
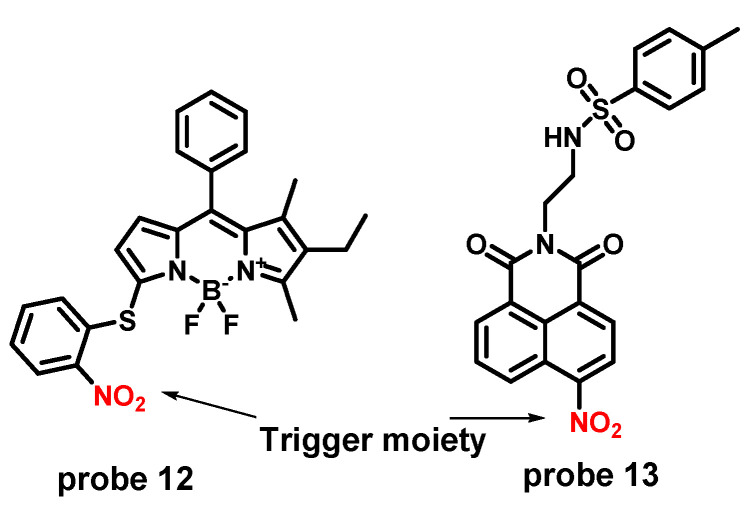
Chemical structures of **probes 12** and **13**.

Some pathogenic bacteria and tumor cells produce high levels of NTR, so developing fluorescence probes targeting NTR for cancer and bacteria imaging is popular [39]. Meanwhile, NTR can catalyze the reduction of nitroaromatic compounds to aromatic amines and as a famous electron-withdrawing group, nitro can quench fluorophore through the PET effect. Therefore, nitro can be modified in the adjacency of fluorophore as a trigger moiety to make NTR probes [40,41] (Figure 4). **Probe 12** was used to imaging NTR in Hela and HepG2 cell and HepG-2 tumor-bearing mice model. **Probe 13** was developed for monitoring NTR in endoplasmic reticulum.

**Figure 5:**
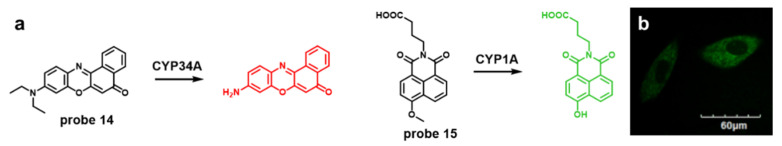
(**a**) Chemical structures of **probes 14** and **15** against CYPs; (**b**) Confocal fluorescence images of A549 cells. Cells incubated with **probe 15** (50 μM) for 1h. The fluorescence images were acquired using 405 nm excitation and fluorescent emission windows: green emission channel. (Reprinted with permission from [46]. Copyright 2015 American Chemical Society.).

Cytochrome P450s (CYPs) are a large enzyme family which includes hundreds of members and they make a vital contribution to the metabolism of toxin, drug and endogenous substrates. Meanwhile, different subtypes of CYPs have different substrate specificity and physiological function. Thus, a series of fluorescence probes for these subtypes of CYPs were developed [42]. Among CYP enzymes, CYP34A is the most abundant and plays a vital role in drug metabolism [43]. A famous fluorescence dye, nile red (**probe 14**), was discovered as a new fluorescent probe for CYP34A [44] (Figure 5a). CPY1A is the other important CYP subfamily and widely involved in bioactivation of procarcinogenic compounds [45]. Therefore, it is highly relevant to oncogenesis. A fluorescent probe, **probe 15**, was reported [46]. It has high specificity for the CPY1A subfamily (Figure 5a) and was applied in imaging CPY1A in cells (Figure 5b). 

**Figure 6:**
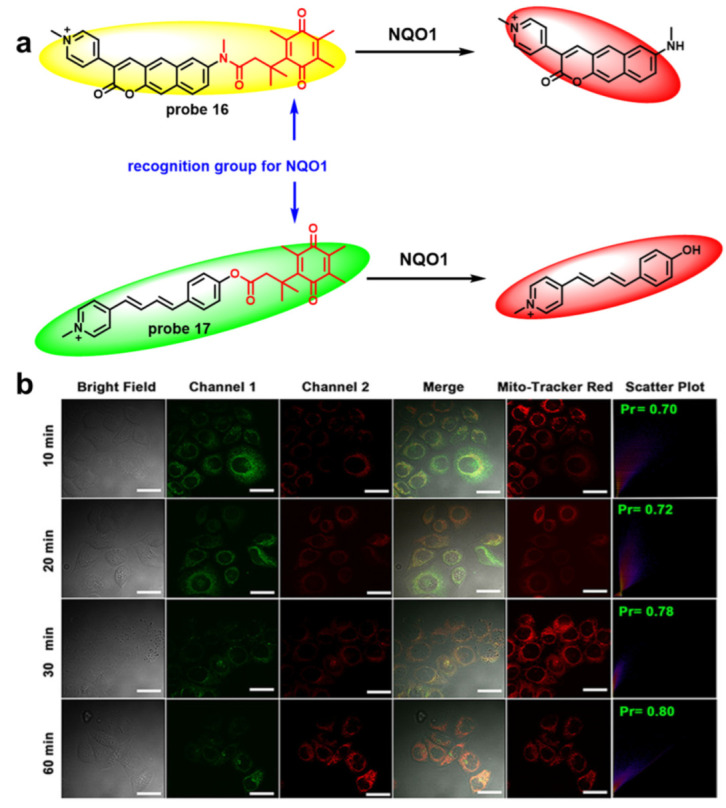
(**a**) Chemical structures of **probes 16** and **17**; (**b**) Confocal fluorescence images of HepG2 cells stained with 10 μM **probe 17** for 10, 20, 30, or 60 min followed by costaining with Mito-Tracker Red (500 nM) for another 0.5 h. Channel 1: λ_ex_ = 790 nm and λ_em_ = 505 ± 25 nm; channel 2: λ_ex_ = 790 nm and λ_em_ = 615 ± 25 nm; Mito-Tracker Red: λ_ex_ = 559 nm and λ_em_ = 590–650 nm. Merge: overlay of channel 1, channel 2, and bright field. Scatter plot: intensity correlation of fluorescence signals in the green channel (channel 1) and the Mito-Tracker Red channel Scale bar = 20 μm. (Reprinted with permission from [51]. Copyright 2021 American Chemical Society.).

NQO1 (NAD(P)H: quinone Oxidoreductase 1) is an important oxidoreductase biomarker. It has several biological functions including the reduction of quinone compounds, maintenance of reduction stress, control of mRNA translation and stabilization of proteins and is related to many diseases, such as cardiovascular diseases, diabetes mellitus, metabolic syndrome, aging, Alzheimer’s disease and cancer [47]. So, lots of fluorescent probes for NQO1 were synthesized for research and diagnosis of these diseases, especially cancer. While quinone propionic amides or acid esters with three methyl groups (in "trialkyl lock" positions) were reduced, a spontaneous lactonization reaction can happen [48]. Meanwhile, the quinone propionic amides or acid esters are easily able to accept electrons from the fluorescence dye in excited state to quench fluorescence through the PET effect. So, it is an excellent recognition group which can be widely used. Based on this recognition group, many fluorescent probes for NQO1 were discovered accordingly [49,50] (Figure 6a). **Probe 16** was a ratiometric fluorescent probe. By reacting with NQO1, its emission wavelength moves to 650 nm from 555 nm. Furthermore, **probe 16** can be used to detect NQO1 in HT-29 cells. **Probe 17** was a ratiometric fluorescent probe as well and can produce the ratio fluorescence response (564/480). Importantly, it has an ultralow response time (4 min) and LOD (0.9 nM) and was used to detect NQO1 in the mitochondria of HepG2 cells (Figure 6b).

## 4. Application of Fluorescent Probes Targeting Enzymes in Fluorescence-Guided Surgery

As a useful detection tool, the fluorescent probe for enzymes can be applied in wide fields, such as inhibitor screening, imaging in cell and living body, biological research and diagnosis. In addition to these familiar applications, it can be used in fluorescence-guided surgery [51]. In cancer surgery, it is not easy for the surgeon to accurately identify the tumor and healthy tissue. Therefore, intraoperative optical fluorescence imaging is a promising method to help doctors [52]. For the fluorescence-guided surgery, an excellent fluorescence imaging agent is necessary. So, a series of fluorescent probes targeting the enzymes with high expression in cancer cells were developed as imaging agents.

**Figure 7:**
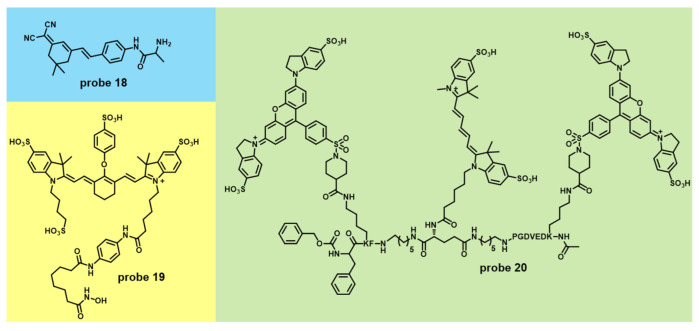
Chemical structures of **probes 18**, **19** and **20**.

The target of **probe 18** is aminopeptidase N (APN), a biomarker of cancer (Figure 7). The probe can lighten the HepG-2 xenograft tumor in BABL/c mice model through in situ spraying manner [53]. Importantly, the method achieved superhigh tumor-to-normal (T/N) tissue ratios (13.86). **Probe 19** is a “always-on” fluorescent probe for Histone deacetylases (HDACs) that were observed to have aberrant overexpression in many cancers [54] (Figure 7 and Table 1). Target-binding affinity of the chemistry probe is stronger (IC_50_ = 196 nM). At 12 h after the probe was injected in the live tumor nude mice model, the tumor was clearly labeled and demonstrated a significant difference from surrounding normal tissues. **Probe 20** can be activated while two cancer biomarker enzymes (cathepsin and caspase 3) all exist [55] (Figure 7 and Table 1). Therefore, the probe can decrease the probability of false positives and be more specific and sensitive to tumor imaging. With the help of the probe, the tumor of the BALB/c ByJ female mice breast cancer model was clearly distinguished and removed.

## 5. Future Development of Fluorescent Probes 

Even though numerous probes have been developed to suit various applications, it is difficult to design and develop an ideal probe for all applications [7]. Additionally, it has been noted that greater photo-stability and brightness, more controlled intracellular localization, and superior absorption, distribution, metabolism, excretion and toxicity (ADMET) properties are expected from next-generation probes. When considering in vivo imaging, fluorescent materials suitable for multiphoton excitation or fluorescence up-conversion are expected to be effective. The other imaging agents such as hybridization of fluorescent probes with other imaging agents, such as those for magnetic resonance imaging and nuclear imaging, have already emerged and are a promising field [56]. Furthermore, it will be important to design probes whose fluorescence intensity lasts for a longer period and suit the biological samples such as serum and urine, which possess a higher amount of background fluorescence. Lanthanide complexes targeting proteases, with extraordinarily long-lived luminescence, have been found to be advantageous in diagnosis of cancer. The basic “skeleton” systems which could be modified to develop novel fluorescent probes may provide a wider advantage. As pointed out by [27], the collaboration of scientists in different fields, from photophysics to clinical pathology, will lead to novel designs and applications of probes and speed up the discovery of fluorescent probes which target enzymes. It will also widen the use of the tools available for further studies of metabolic processes mediated by a large collection of enzymes in living cells. With the deepening of modern research, more and more bioactive molecules have been discovered. People urgently want to understand the existence state and action mechanism of various bioactive molecules in the process of various life activities, and a fluorescence probe is an indispensable tool. Through the use of fluorescent probes, people can obtain a more intuitive understanding of the variety of the mysteries of life activities. As an overall note, the fluorescent probes targeting enzymes is an interesting and continuously improving area for the study of enzyme behavior in living systems.

## Figures and Tables

**Table 1:** The basic information of listed probes in the main text.

NO	λ_ex_ (nm)	λ_em_ (nm)	LOD	Target	Application	Ref.
**probe 1**	340	505	68 ng/mL	elastase	inhibitor screening	[23]
**probe 2**	670	700	29.6 ng/mL	elastase	cell and mice imaging	[22]
**probe 3**	490	520	/	chymotrypsin	clinical detection	[24]
**probe 4**	670	695	13 mU/mL	chymotrypsin	cell and mice imaging	[25]
**probe 5**	520	560	0.36 U/mL	AChE	cell and mice imaging	[27]
**probe 6**	670	700	117.3 mU/mL	AChE	cell and zebrafish imaging	[26]
**probe 7**	455	515	/	BChE	inhibitor screening and cell imaging	[28]
**probe 8**	665	705	/	BChE	cell, zebrafish and mice imaging	[29]
**probe 9**	390	470	/	BACE1	inhibitor screening and cell imaging	[33]
**probe 10**	720	750	/	MMPs	cell and mice imaging	[35]
**probe 11**	405	465 and 665	/	Caspases	cell imaging	[37]
**probe 12**	450	505	22 ng/mL	NTR	cell and mice imaging	[40]
**probe 13**	440	543	36 ng/mL	NTR	cell and tissue slices imaging	[41]
**probe 14**	470	570	/	CYP34A	/	[44]
**probe 15**	452	564	0.02 nmol/mL	CYP1A	cell and tissue slices imaging	[46]
**probe 16**	405	650/555	4.99 μg/mL	NOQ1	cell imaging	[50]
**probe 17**	407	564/480	0.9 nM	NQO1	cell and mice imaging	[49]
**probe 18**	445	650	0.13 ng/ml	APN	cell imaging and fluorescence-guided surgery	[53]
**probe 19**	775	801	/	HDACs	fluorescence-guided surgery	[54]
**probe 20**	640	670	/	cathepsin and caspase 3	cell, mice imaging and fluorescence-guided surgery	[55]
